# Predictors of mortality among multidrug-resistant tuberculosis patients in central Ethiopia: a retrospective follow-up study

**DOI:** 10.1017/S0950268820002514

**Published:** 2020-10-15

**Authors:** Getahun Molla Kassa, Abilo Tadesse, Yalemzewod Assefa Gelaw, Temesgen Tadesse Alemayehu, Adino Tesfahun Tsegaye, Koku Sisay Tamirat, Temesgen Yihunie Akalu

**Affiliations:** 1 Department of Epidemiology and Biostatistics, Institute of Public Health, College of Medicine and Health Science, University of Gondar, Gondar, Ethiopia; 2 Department of Internal Medicine, School of Medicine, College of Medicine and Health Science, University of Gondar, Gondar, Ethiopia; 3 Population Child Health Research Group, School of Women's & Children's Health, UNSW Sydney, Sydney, Australia; 4 Department of Radiology, School of Medicine, College of Medicine and Health Science, University of Gondar, Gondar, Ethiopia; 5 Department of Epidemiology, University of Washington, Seattle, Washington, USA

**Keywords:** Follow-up study, mortality, multidrug-resistance tuberculosis, predictors

## Abstract

The burden of multidrug-resistant tuberculosis (MDR-TB) related to mortality in resource-poor countries remains high. This study aimed to estimate the incidence and predictors of death among MDR-TB patients in central Ethiopia. A retrospective follow-up study was conducted at three hospitals in the Amhara region on 451 patients receiving treatment for MDR-TB from September 2010 to January 2017. Data were collected from patient registration books, charts and computer databases. Data were fitted to a parametric frailty model and survival was expressed as an adjusted hazard ratio (AHR) with a 95% confidence interval (CI). The median follow-up time of participants was 20 months (interquartile range: 12, 22) and 46 (10.20%) of patients died during this period. The incidence rate of mortality was 7.42 (95% CI 5.56–9.91)/100 person-years. Older age (AHR = 1.04, 95% CI 1.01–1.08), inability to self-care (AHR = 13.71, 95% CI 5.46–34.40), co-morbidity (AHR = 5.74, 95% CI 2.19–15.08), low body mass index (AHR = 4.13, 95% CI 1.02–16.64), acute lung complications (AHR = 4.22, 95% CI 1.66–10.70) and lung consolidation at baseline (AHR = 5.27, 95% CI 1.06–26.18) were independent predictors of mortality. Most of the identified predictor factors of death in this study were considered to be avoidable if the TB programme had provided nutritional support for malnourished patients and ensured a close follow-up of the elderly, and patients with co-morbidities.

## Introduction

Tuberculosis (TB) causes inestimable suffering and claims millions of lives worldwide. Multidrug-resistant tuberculosis (MDR-TB), defined as resistance to at least two first-line anti-TB drugs (isoniazid and rifampicin), has become a significant threat to global public health [1]. The extent and burden of MDR-TB vary significantly but the burden of disease is highest in resource-poor countries [2, 3]. Patients who have a previous history of TB treatment present a higher risk of developing MDR-TB, indicating the failure of TB control programmes [4, 5]. In 2018, an estimated half a million new cases of rifampicin-resistant (RR-TB) and MDR-TB worldwide with a prevalence varying from 3.4% to 18% among new and previously treated cases respectively [1]. Globally, 30 countries accounted for more than 90% of the MDR-TB cases [6], and more than half originated from Russia, India and China [1]. In Africa, the MDR-TB prevalence was estimated to be 2.5% and 12% among new and re-treatment cases, respectively [1]. Ethiopia is one of the 27 high MDR-TB burden countries and ranks third in the continent with an estimated 2100 annually notified MDR-TB cases [7, 8]. The prevalence of MDR-TB in Ethiopia reported in 2018 was 0.71% among new, and 16% among re-treatment cases [1]. There have been progressive improvements worldwide in the scaling up of MDR-TB case detection and treatment enrolment in recent years [6]. In Ethiopia, the Programmatic Management of Drug-resistant TB (PMDT) was started in 2009 in response to the first 45 MDR-TB patients identified at St. Peter's TB Specialist hospital [9]. Globally, as of 2018, the World Health Organisation (WHO) has reported that nearly 50% of MDR-TB patients were successfully treated but there were still high rates of mortality and loss of patients to follow-up [1]. Overall, the high death and default rates are generally cited as the major obstacles in achieving better cure rates [1, 10, 11]. As the death of a patient during TB treatment signifies a failure of the programme, the accurate determination of the cause of death is the first step towards designing appropriate and timely interventions to prevent premature deaths. Identifying and effectively addressing risk factors for death among MDR-TB patients is therefore necessary to improve treatment outcomes. As such information remains limited, this study aimed to identify the rate and predictors of mortality among MDR-TB patients in the Amhara region of Ethiopia through retrospective survey data from September 2010 to December 2017.

## Methods

### Study design and setting

A retrospective study was conducted in three selected treatment-initiated centre hospitals of the Amhara region, namely the University of Gondar, Borumeda, and Debre-Markos Referral Hospital which together provide clinical services for approximately 90% of MDR-TB patients in the region. All isolates from TB-positive individuals were screened for rifampicin resistance at baseline using the rapid drug susceptibility testing (DST) technique in the GeneXpert system (manufactured by Cepheid, located in California, America) or the line probe assay (LPA) (manufactured by the Hain Lifescience GmbH based in Nehren in the district of Tübingen, Germany). Isolates from all patients with confirmed RR/MDR-TB were tested using the LPA for core second-line drugs before or within 1 week of treatment initiation with the MDR-TB regimen. All patients with isolates resistant to first- and second-line anti-TB drugs were further evaluated using culture and phenotypic DST [7]. All patients with MDR-TB were treated in the hospital setting under medical supervision. Patients requiring treatment at the beginning or any stage of care were admitted to the hospital. The recommended commonly used drugs for the treatment of MDR-TB in Ethiopia were bedaquiline (Bdq), levofloxacin (Lfx), moxifloxacin (Mfx), linezolid (Lzd), clofazimine (Cfz), cycloserine (Cs), amikacin (Am), capreomycin (Cm), delamanid, prothionamide (Pto), pyrazinamide (Z), isoniazid high dose (H^HD^) and ethambutol (E). Three MDR-TB treatment regimens in Ethiopia are recommended based on patients' clinical and laboratory characteristics. The first is the all-oral longer regimen of Bdq-Lfx-Lzd-Cfz-Cs for 18–20 months; the second is a shorter regimen of Am-Mfx-Pto-Cfz-Z-H^HD^-E/5 Mfx-Cfz-Z-E for 9–12 months. The last is an individualised regimen comprising at least 4–5 likely effective drugs based on their availability, the susceptibility of the isolate, and clinical expert decision for 18–24 months. This regimen is tailored to the specific case scenario of each patient.

### Data collection and variables of the study

Time to death was the response variable; explanatory variables were socio-demographic characteristics (sex, age, residence, marital status, occupation and educational status), behavioural factors (smoking and alcohol drinking), clinical characteristics (baseline body mass index (BMI), HIV co-infection, MDR-TB category, co-morbidities, lung complications, radiological findings, time of sputum culture conversion, number of previous TB treatments, TB type and functional status).

Death was defined as a documented death of a patient while on MDR-TB treatment. Patients who were cured, treatment completed, treatment failed, lost to follow-up, transferred out and on treatment at the end of the study, were considered as censored. A new TB case was defined as a patient who had no prior anti-TB treatment or had been treated for less than 1 month. A previously treated case was one who had been treated for TB for more than 1 month, other than for chemoprophylaxis. BMI was defined as low if <18.5 kg/m^2^ and normal if ⩾18.5 kg/m^2^. The ability to self-care was a patient who was ambulant and able to perform daily activities; patients unable to meet these criteria were categorised as lacking self-care.

Sputum culture conversion was evidenced by two consecutive TB- negative cultures sampled at least 30 days apart; the date of the first sample was taken as the date of conversion. Two consecutive positive cultures, taken at least 30 days apart, represented ‘culture reversion’ [7]. WHO clinical stages 3 and 4 were considered as a marker of advanced severe TB or advanced HIV [12].

Patients and the public were not involved in the design and conception of the study and it was not possible to communicate the results to patients owing to the retrospective nature of the study.

### Data collection and analysis

Data were collected using a data extraction check-list. This was prepared in English after reviewing the treatment registration logbook and patient follow-up charts, as well as computer databases. After checks for consistency and completeness, data were entered into Epi-data version 3.1 and exported to Stata version 14.0 for analysis. Summary statistics were used to describe demographic, behavioural and clinical data. The incidence rate was calculated by dividing the number of total deaths by the total person-years of the follow-ups. The life table and Kaplan–Meier curves were used to estimate the overall survival time and supplemented by a log-rank test to compare the survival curves of different exposure groups. Model comparison was performed using the smallest values of the Akaike information criterion and Bayesian information criterion. Both bi-variable and multivariable Weibull regression with the gamma frailty model was fitted to identify predictors of mortality. Variables with a *P*-value <0.2 in the bi-variable analysis were fitted to the multivariable analysis, and variables <0.05 in the latter analysis were considered statistically significant.

## Results

### Baseline socio-demographic and behavioural characteristics

A total of 451 patient records from the three referral hospitals were eligible for analysis. Of these, 58.54% were males; the mean patient age was 31.29 (±12.02) years, 80.49% were Orthodox Christians followed by Muslims (18.18%). The great majority (94.24%) were from Amhara Regional State most of whom (51.22%) were rural dwellers (Table 1).

**Table 1.** Baseline socio-demographic and behavioural characteristics of MDR-TB patients in the Amhara region, September 2010–January 2017

Variable	Number	Percentage
Sex		
Male	264	58.54
Female	187	41.46
Age in years		
⩽24	151	33.48
25–34	140	31.04
35–44	86	19.07
⩾45	74	16.41
Marital status		
Married	194	43.02
Single	157	34.81
Divorced	54	11.97
Widowed	10	2.22
Separated	36	7.98
Level of education		
Not formally educated	186	41.52
Primary	140	31.25
Secondary	78	17.41
Tertiary	44	9.82
Residence		
Urban	219	48.67
Rural	231	51.33
Occupation		
Not employed	183	41.6
Employed	268	58.4
Smoking		
No	391	86.89
Yes	59	13.11
Alcohol use		
No	362	80.44
Yes	88	19.56

### Baseline clinical characteristics

The majority (92%) of patients presented with pulmonary TB. Most (72%) of the participants had a baseline BMI of <18.5 kg/m^2^ 94% of patients had a history of prior TB treatment, most of whom (62.3%) had received first-line anti-TB drugs on more than one occasion. One-hundred and eighteen of 451 patients (26.2%) were HIV positive, with a median CD4 count of 179 (interquartile range (IQR): 91, 279), and 63.9% were in WHO clinical stage 3 at the commencement of MDR-TB treatment. Of the total HIV co-infected patients, 108 (91.5%) were on antiretroviral treatment (ART) at the beginning of the MDR-TB treatment. A total of 367 patients had baseline chest X-rays of which 49.9% and 34.1% had evidence of cavitation and infiltration, respectively (Table 2).

**Table 2.** Clinical characteristics of MDR-TB patients at the University of Gondar Comprehensive Specialised Hospital, September 2010–January 2017

Variable	Frequency	Percentage
BMI		
<18.5	321	71.49
⩾18.50	128	28.51
Functional status		
Can care self	371	82.26
Can't care self	80	17.74
No of previous TB treatment		
<2	160	35.48
⩾2	291	64.52
Acute lung complication		
Pneumonia	47	10.42
Pneumothorax	11	2.44
Chronic lung complication		
Cor pulmonale	6	1.33
Bronchiectasis	3	0.67
Chronic obstructive pulmonary disease (COPD)	16	3.55
Fibrotic change	25	5.54
Co-morbidity		
No	406	90.83
Yes^a^	41	9.17
HIV^b^ co-infection		
No	328	73.54
Yes	118	26.46
Radiologic finding		
Cavity	183	49.86
Multilobal and bilateral Consolidation	56	15.26
Infiltration	125	34.06
Chronic change	99	26.98
Effusion	34	9.26
Others^c^	29	7.9

a Diabetes mellitus, hypertension, bronchial asthma, COPD, chronic kidney disease, epilepsy, cardiac diseases, deep venous thrombosis and gangrene, depression, visceral leishmaniasis, toxic goitre, rheumatoid arthritis, cholelithiasis, cervical cancer and glaucoma.

b HIV (human immunodeficiency virus).

c Hilar lymphadenopathy, normal, cardiomegaly, empyema and fungal ball.

### Time to death of MDR-TB patients on treatment

**Fig. 1.**
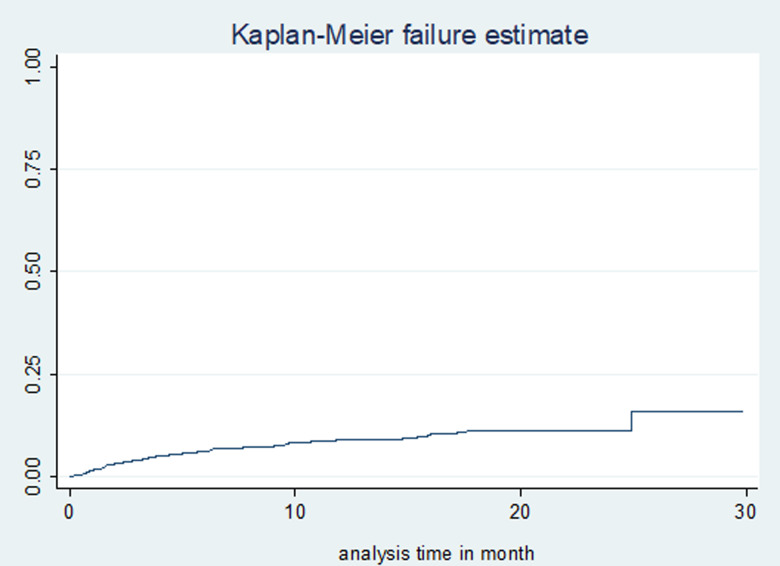
Kaplan–Meier failure (death) estimates of MDR-TB treatment in Amhara Regional State, September 2010–January 2017.

**Fig. 2.**
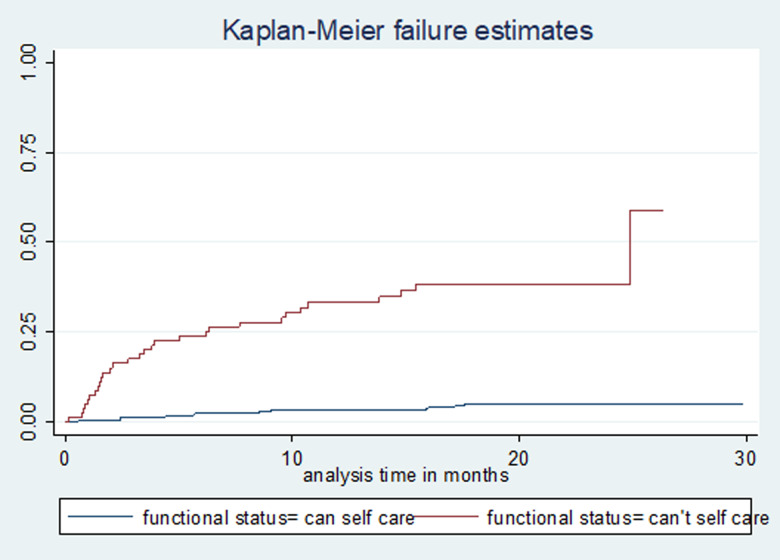
Kaplan–Meier failure (death) estimate curve by functional status in Amhara Regional State, September 2010–January 2017.

Study participants were followed for a total of 7541 person-months (628 person-years), with a median follow-up time of 20 months (IQR: 12, 22). A total of 230 (51%) patients were cured, 22 (4.9%) completed treatment, 46 (10.2%) died, 32 (7.1%) were lost to follow-up, 105 (23.38%) remained on treatment, 12 (2.7%) were transferred out and 4 (0.9%) had treatment failure. The incidence rate of mortality was 7.42 (95% confidence interval (CI) 5.56–9.91) per 100 person-years observation. The cumulative probability of death at the end of 6 months, 1 year, 2 years and 2.5 years were 6.2% (95% CI 4.31–8.9), 9.0% (95% CI 6.65–12.2), 11.1% (95% CI 8.41–14.6) and 14.8% (95% CI 8.74–24.56), respectively (Fig. 1). The mean survival time for patients who couldn't self-care was 14 months, compared with 26 months for those who could self-care at the time of death; the difference was strongly significant (*P*-value <0.0001) (Fig. 2).

### Predictors of mortality

The bi-variable Weibull regression with gamma frailty analysis showed that age, sex, BMI, inability to self-care, acute and chronic lung complications, occupation, functional status, co-morbidity, consolidation and effusion on baseline chest X-ray, were associated with mortality. However, in the multivariable model, advanced age, patients who couldn't self-care, presence of co-morbidity, BMI < 18.5 kg/m^2^, acute lung complication and multi-lobar and bi-lateral consolidation remained significant predictors of mortality (Table 3).

**Table 3.** Bi-variable and multivariable Weibull regressions with gamma frailty model for predictors of death among MDR-TB patients at Amhara Regional State, September 2010–January 2017

Variable	Mortality status		CHR (95% CI)	AHR (95% CI)
	Event	Censored		
Age in years	31.29 (±12.02)	1.07 (1.02, 1.12)	**1.04 (1.01–1.08)**	
Sex				
Male	22	242		
Female	24	163	2.45 (0.83–7.26)	1.19 (0.47–3.03)
BMI				
<18.5 kg/m^2^	39	282	8.44 (1.28–14.48)	**4.13 (1.02–16.64)**
⩾18.50 kg/m^2^	7	121	1	
Functional status				
Can care self	16	355	1	
Can't care self	30	50	32.26 (12.76–81.54)	**13.71 (5.46–34.40)**
Chronic lung complication				
No	32	54		
Yes	14	356	5.14 (1.41–18.73)	1.673 (0.66–4.21)
Acute lung complication				
No	25	35		
Yes	21	370	13.99 (5.34–36.64)	**4.22 (1.66–10.70)**
Co-morbidity				
No	31	375	1	
Yes	14	27	14.52 (4.00–52.69)	**5.74 (2.19–15.08)**
Occupation				
Employed	26	242		
Unemployed	20	163	3.00 (0.94–9.54)	1.04 (0.34–3.17)
Radiologic finding				
Multilobar and bilateral consolidation				
No	43	352		
Yes	3	53	4.34 (0.78–24.05)	**5.27 (1.06–26.18)**
Effusion				
No	38	379		
Yes	8	26	3.46 (0.69–17.33)	2.13 (0.64–7.09)

As age increased by 1 year the hazard of death was increased by 4% (adjusted hazard ratio (AHR) = 1.04, 95% CI 1.01–1.08). Patients who couldn't self-care had a 14.71-fold higher hazard of death compared to those who could self-care (AHR = 13.71, 95% CI 5.46–34.40). For patients with co-morbidities, the hazard of death was 5.74-fold higher than those who had no co-morbid factors (AHR = 5.74, 95% CI 2.19–15.08). Similarly, a BMI < 18.5 kg/m^2^ was associated with a 4.13-fold hazard increase compared to those above 18.5 kg/m^2^ (AHR 4.13, 95% CI 1.02–16.64); acute lung complications were 4.22-fold higher compared with others (AHR 4.22, 95% CI 1.66–10.70) and those patients with multi-lobar and bilateral consolidation had a 5.27-fold higher hazard of death compared with no consolidation (AHR 5.27, 95% CI 1.06–26.18).

## Discussion

In this study, the incidence of mortality was 7.42 (95% CI 5.56–9.91)/100 person-years follow-up during treatment and most patients died early on the initiation of treatment. This finding accords with earlier reports from Ethiopia [13, 14] but our incidence was considerably higher than that found in Eastern Europe (1.094/100 person-years) [15]. This might be due to the early detection of TB cases in Europe (46%), than in Ethiopia (8.6%). Several factors, no doubt, contribute to the latter statistic and include better health care systems with advanced supportive patient management, higher socio-economic status and fewer co-morbidities such as malnutrition and HIV in Europe. The higher death rate in Ethiopia might also be attributed, in part, to the constitution, and duration, of the current treatment regimen used in Ethiopia. In particular, the use of drugs such as Mfx have reported higher efficacy for TB in Europe compared to the lower efficacy of Lfx, commonly prescribed and used in Ethiopia. Indeed, some evidence suggests that patients treated with Mfx have a better treatment outcome and earlier conversion to negative cultures [16, 17]. There is no comprehensive DST programme for first- and second-line TB drugs in the Amhara region; consequently, all patients are put on standardised regimens (i.e. constructed for the majority of patients) instead of tailored regimens based on susceptibility test results of individual patients which have been shown to achieve more successful outcomes [18]. Moreover, putting MDR-TB patients for more than 20 months on treatment would likely increase severe adverse events which can contribute additionally to the risk of death [19]. A recent study in our region showed the median time to commencement of treatment after a diagnosis of MDR-TB was 8 days [20] which falls short of international standards in developing countries, and so may play a role in the high death rate [21]. A number of other factors including older age, inability to self-care, co-morbid conditions, acute lung complications, low BMI and multi-lobar or bilateral lung consolidation also contributed to mortality.

Our finding of an association between increasing age and death has been noted by a number of studies across the world [15, 22–24]. This could be linked to the risk of chronic diseases in the elderly and general physical deterioration along with impaired immunity [23, 24]; the latter has been associated with high mortality among MDR-TB patients [25, 26]. Other contributory factors may include the reluctance of the elderly to consult healthcare facilities leading to delayed presentation and diagnosis compared with younger patients [25, 26].

Patients who couldn't care for themselves were at a higher risk of mortality than those who could. This finding was consistent with a study from Russia [27] and was not surprising as such patients could be bedridden with other advanced diseases and are also likely to be on several medications which could result in drug interactions and poor compliance with anti-TB medication due to a high pill burden. The severity of TB may also result from infection with highly virulent strains, suboptimal host response and/or extended disease duration culminating in a significantly higher risk of death [28]. It is widely recognised that the management of MDR-TB in critically sick patients is challenging and compounded by pharmacokinetic issues such as poor gastric absorption, high rates of organ dysfunction and drug toxicity [29] often necessitating admission to intensive care [13], which are lacking in our study region. Alcohol abuse has long been associated with poor TB treatment outcomes [30]. A number of patients who couldn't self-care also consumed alcohol in this study. Furthermore, the majority were co-infected with HIV, had low BMI, increased co-morbidities and clinical complications all of which would accelerate their mortality [13, 22]. Medications given for co-morbid conditions may interact with MDR-TB drugs and overlap in toxicity, often leading to poor compliance by patients [13, 31]. For example, the management of MDR-TB is similar for both diabetic and non-diabetic patients and it is recognised that diabetes patients have worse TB treatment outcomes due to the induction of poor glucose control by TB itself [32]. Additionally, glucose control drugs such as metformin may induce more pronounced gastrointestinal side-effects when co-administered with the anti-TB agents ethionamide and *para*-aminosalicylic acid [33].

HIV co-infection is not associated with mortality in our study. The possible explanation might be the integration of TB-HIV service and all of our patients are on ART. Studies also show response to DR-TB treatment did not differ with HIV infection with access to and earlier ART, which resulting in the slow progression of DR-TB and limiting with advanced HIV [23, 34].

Patients with acute lung complications had a higher risk of death as a consequence of infection of the lower respiratory tract leading to the accumulation of inflammatory exudate in the alveoli resulting in reduced oxygen exchange and respiratory insufficiency. MDR-TB patients with clinical complications also experience longer recovery times and poor response to TB-medications [31, 35]. The relationship between a low BMI and risk of death has been noted in several studies worldwide [24, 28, 34, 36–38] and has been strongly linked to poor nutritional status, which was evident in over 70% of our patient cohort. To address this issue all patients in Amhara region received food packages obtained from the global fund but they are not very adequate. Economical support and early nutritional intervention has been shown to reduce the mortality of MDR-TB patients [28, 39], and in resource-limited settings, profound undernutrition and gastrointestinal toxicities have been noted during MDR-TB treatment [37]. Malnutrition is also a predisposing factor for impaired host immunity to TB. Such patients might also lack essential micronutrients rendering them more susceptible to life-threatening diseases [24, 34]. It follows that weight gain is a useful interim marker for favourable TB treatment outcomes [38].

We found that patients with pulmonary consolidation at baseline were at a greater risk of death than those without consolidation, a finding previously reported from China [36]. The presence of a multi-lobar and bilateral consolidation on a chest X-ray shows a severely damaged lung due to which the patient may not be able to get enough oxygen that can cause damages to other body organs. As the penetration of drugs into highly damaged lungs is poor, patients do not respond to treatments and the infection worsens with a higher hazard of death. The other possible explanation is the lack of surgery for patients having cavitary and or lobar consolidation, with optimal medical follow-up and therapy in the management of MDR-TB in the Amhara region. The WHO 2018, PMDT guideline indicates that MDR-TB patients with surgical management appear to have a superior outcome, lower mortality rate and reduced recurrences compared with medical therapy alone [40].

The key strength of this study was the incorporation of a multi-centre site and employing an appropriate statistical model for better estimation of risk factors. However, some important variables such as the specific cause of death, patient compliance with therapy and knowledge of the drug-resistance patterns of patient isolates are lacking due to the nature of the data collected during routine clinical practice. The 105 participants classified as on-treatment had no equal follow-up time as for the remainder of MDR-TB patients and were included in the final regression analysis thus potentially lowering the number of deaths which may impact the HR. The overall mortality rate in this study population was high making it difficult to meet the target of the end-TB strategy aimed at reducing 95% of TB-related mortality by 2035.

In conclusion, the lack of data on the specific cause of death brings into question whether the factors predictive of death are indeed linked to MDR-TB or other variables. Except for age many of the independent risk factors identified were modifiable. It is, therefore, time to think about nutritional support, early detection and management, and shorten the duration of the MDR-TB treatment regimen to improve the chances of meeting the goal of markedly reducing the incidence and mortality of TB in Ethiopia.

## Data Availability

Data will be available upon a reasonable request from the corresponding author.
